# Facilitating home birth in perinatal palliative care: A case report

**DOI:** 10.1177/02692163241280374

**Published:** 2024-09-27

**Authors:** Sophie Bertaud, Rachel Kirven, Thomas Kirven, Emily Harrop, Amanda Crudgington, Dominic Wilkinson

**Affiliations:** 1 Oxford Uehiro Centre for Practical Ethics, Faculty of Philosophy, University of Oxford, Oxford, UK; 2 Louis Dundas Centre for Children’s Palliative Care, Great Ormond Street Hospital for Children, London, UK; 3 Ethox Centre, Nuffield Department of Population Health, University of Oxford, Oxford, UK; 4 Independent contributor, Oxford, UK; 5 Helen & Douglas House, Oxford, UK; 6 John Radcliffe Hospital, Oxford, UK; 7 Florence Park Midwives Team, Maternity Department, John Radcliffe Hospital, Oxford, UK; 8 Murdoch Children’s Research Institute, Melbourne, VIC, Australia; 9 Centre for Biomedical Ethics, National University of Singapore Yong Loo Lin School of Medicine, Singapore, Singapore; 10 Department of Paediatrics, School of Clinical Sciences, Faculty of Medicine Nursing and Health Sciences, Monash University, Melbourne, VIC, Australia

**Keywords:** Pregnancy, palliative care, perinatal care, infant, newborn, hospice and palliative care nursing, midwifery

## Abstract

**Background:**

Perinatal palliative care can offer compassionate support to families following diagnosis of a life-limiting illness, to enable them to make valued choices and the most of the time that they have with their newborn. However, home birth is usually only offered in low-risk pregnancies.

**Case:**

A couple who received an antenatal diagnosis of hypoplastic left heart syndrome and who had made a plan to provide palliative care to their baby after birth requested the option of a home birth.

**Possible courses of action:**

Recommend birth at hospital or explore the possibility of a home birth with perinatal palliative care support.

**Formulation of a plan:**

Multidisciplinary discussion and collaboration enabled a plan for home birth to be made which anticipated potential complications.

**Outcome:**

The baby was born at home and died on day 5 of life receiving outreach nursing, paediatric and palliative care support and buccal and oral opioids for symptom management. We include reflections from the family on the importance of this experience.

**Lessons:**

We provide a list of potential criteria for considering home birth in the setting of perinatal palliative care.

**View:**

Facilitating a home birth in the setting of perinatal palliative care is an option that can be hugely valued by families, but this service may be practically difficult to deliver in many contexts. Further research is needed to understand the preferences of women and families receiving perinatal palliative care.

**What is already known about this topic**

- Facilitating choices for families, such as place of birth and place of death, is an important element of perinatal palliative care
- The diagnosis of a serious condition in a fetus would usually preclude birth taking place outside of a hospital setting

**What this paper adds?**

- Facilitating a home birth in the context of perinatal palliative care is possible in selected cases
- Experiencing a home birth when a baby is expected to die can be hugely valuable for some families
- Delivering this type of care safely requires adequate resources to allow for forward planning and coordination of care in the community

**Implications for practice, theory or policy**

- Further research is needed to understand the birthing preferences of women and families receiving perinatal palliative care across different global settings
- Healthcare organisations may benefit from multidisciplinary discussions to assess the safety, acceptability and feasibility of providing home births alongside palliative care in their context

## Background

Perinatal palliative care offers holistic support to babies and families when a potentially life-limiting diagnosis is made before or shortly after birth.^1^ There is increasing research evidence to support the benefits of palliative care support throughout pregnancy, birth and the neonatal period for babies with life-limiting conditions and their families^2^ but such services are absent or patchy in many countries and perinatal palliative care remains poorly described in global resource-constrained settings.^3^

A key component of perinatal palliative care is to provide support from the antenatal period onwards and to facilitate important choices for families, including place of birth and death.^4^ In high-resource countries (HRCs), where midwifery services are well-integrated into the health system model of care, giving birth at home is typically a choice (with between 1% and 16% of childbearing people choosing to give birth at home^5^) whereas in low- and middle-resource countries (LMRCs), birth at home occurs much more frequently and may be associated with high mortality, although the number of facility-based births is increasing.^6^ In the UK where healthcare is provided by the publicly-funded National Health Service and is free at the point of use, guidelines from the National Institute for Health and Care Excellence (NICE)^7^ recommend that pregnant people at low risk of complications during labour are given a choice of birth settings including the option of a home birth. However, the diagnosis of a significant fetal abnormality would ordinarily prompt a recommendation for birth to take place at an obstetric unit.

Whilst the choice to pursue a home birth is generally less well supported in the United States,^8^ we found one conference abstract reporting a home birth supported by a home hospice team in Chicago, USA.^9^ We found no other published reports in the international literature of home births in the setting of perinatal palliative care.

## Case

Thomas and Rachel were told at their 20-week anomaly scan that their daughter Lily was affected by hypoplastic left heart syndrome (HLHS), a severe form of congenital heart disease. Parents are routinely offered three options: three-staged reconstructive surgical technique, termination of the pregnancy or compassionate supportive therapy only.^10^ All surgical options are non-curative or ‘palliative’ and carry significant risks of mortality and long-term morbidity.^11^ Thomas and Rachel elected to continue their pregnancy with a plan to provide palliative care to their baby after birth. From early on in the pregnancy, Rachel expressed a desire for a home birth. She had a history of two previous vaginal deliveries without complications.

## Possible courses of action

- Recommend birth at hospital (either on labour ward or a midwife-led birthing unit)
- Explore the possibility of a home birth with perinatal palliative care support

## Formulation of a plan

Through a series of multidisciplinary discussions, plans were made to facilitate birth at home with support from the community midwives, consultant neonatologist and children’s palliative care team. Rachel and Thomas were fortunate to have access to two community midwives who were able to provide continuity of care throughout their pregnancy and delivery and, in addition, they chose to employ a private birth doula who worked closely with the midwifery team. Interprofessional discussions explored the views of all involved and potential for concerns about providing care in an unfamiliar setting. Peer review of the case was sought at a national professional forum for perinatal palliative care. An anticipatory symptom management plan was written to ensure that medications were available in advance of the birth. One practical challenge was that prescription and preparation of medications was not possible prior to birth since a unique medical record number was unable to be assigned.

Parallel plans explored parental wishes in the event of complications of home birth, need for transfer to hospital, difficulties with symptom management, short or longer survival and the possibility of changing parental wishes after birth.

## Outcome

After spontaneous onset of labour at 40 weeks’ gestation, Lily was born at home with two community midwives in attendance who already knew the family well. She was assessed shortly after birth by a consultant paediatrician and at subsequent daily home visits by the neonatal and paediatric palliative care teams and community nursing. She established breast feeding and was initially asymptomatic apart from intermittent grunting and mild cyanosis. On day 3/4, Lily had developed increased work of breathing, and received oral morphine and buccal diamorphine with apparent effect. She appeared comfortable, though fed less frequently and had episodes of colour change. On day 5 in the evening, she had a long apnoea followed by gasping respiration. She received intermittent buccal diamorphine and died in her parents’ arms 2 hours later. Box 1 contains Rachel’s personal account of what a home birth meant to her.

**Box 1.** Rachel’s account of what a home birth meant to her.

When we heard about Lily’s HLHS diagnosis, it was easy to feel like, on a grand scale, so much of our life was out of our control. We soon saw that while living with anticipatory grief was heavy, we also had the gift of time. We could prepare for elements of her birth, life and death that would honour her and our family and help us open to grief. When my request for home birth was considered, I felt an immense sense of relief. I wanted to birth where I felt most comfortable. My husband and I wanted our other two children to be involved in Lily’s life. We longed for memories at home with Lily and for her to have a life with us before she died. My desire was for this time to be characterised by peace and love. While I knew that home birth didn’t necessarily guarantee all this, it felt like a step closer towards these hopes. And it was something I could influence amidst deep pain. Being supported in birthing at home encouraged me to not fear the uncertainty around Lily’s life and death. I wanted to birth Lily to life and also take responsibility for ‘birthing’ her towards death. The support and respect we received from our multiple medical teams made me feel empowered and equipped towards this end. I felt a strong sense of autonomy in how I birthed for and how we cared for Lily. Our children have memories from Lily’s first minutes of life and feel deeply like she is part of our family. This has been instrumental in moving through our grief. I do not feel like this experience happened to us; instead, our family and community were a part of it all from the beginning. I am grateful to have the memories of birthing Lily in our home. I am grateful to have held her warm body against mine on the same sofa where I held my other babies postpartum. I feel privileged to have moments of beauty with her before she died, we said goodbye, and carried her body over the threshold of our home into the care of the hospice team. I will forever treasure the fullness of being with Lily through life and death.

**Figure 1.**
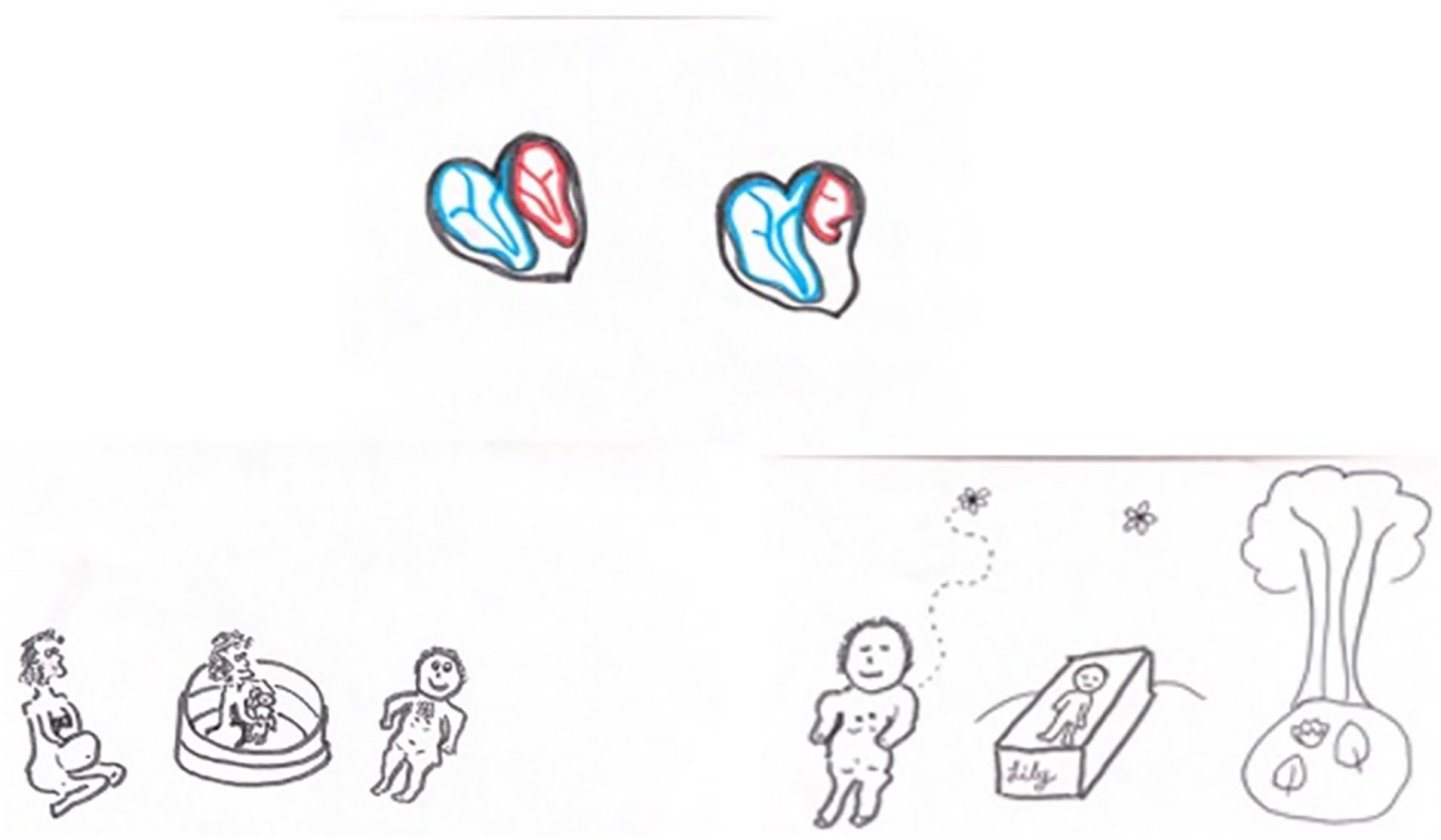
Lily’s story. Pictures drawn by Thomas for Lily’s siblings.

Thomas and Rachel’s older children aged 3 and 5 were present throughout labour and delivery and took an active role in caring for their younger sibling. During pregnancy Thomas and Rachel had spoken to the children about what to expect when their sister was born and used drawings and art activities with the children as a way of facilitating these conversations (Figure 1).

## Lessons

Planned home birth is an important option for a significant number of women. In low-risk pregnancies, it is associated with fewer interventions during labour and for multiparous women there is no impact on perinatal outcomes.^12^ However, compared with ‘low risk’ women planning home birth, ‘higher risk’ women who plan a home birth have a significantly increased risk of an adverse perinatal outcome.^13^ The ‘risk’ of a pregnancy might arise from factors in either the fetus, in the mother or both.

Home birth might be challenging in some cases of antenatally diagnosed severe fetal abnormalities – particularly where prognosis is uncertain, or where there is uncertainty about the appropriateness of palliative care.^14,15^ In a number of cases where perinatal palliative care is offered, parallel planning includes assessment of the infant’s condition at delivery and the option of some acute neonatal interventions. Delivery at home might make it difficult to provide these. In other cases, where an infant is anticipated to have early distressing symptoms (for example respiratory distress or pain), it may be challenging for midwives to support (since such neonatal symptom management is outside their usual scope of practice). In all cases where home birth is being considered, this is likely to require a significant amount of forward planning and access to the staff resources necessary to facilitate planning, coordination and delivery of this level of care. Good practice should include provision of a symptom management plan with the support of a specialist pharmacist with expertise in paediatric palliative care to facilitate both prescription and dispensing of medications at neonatal doses in the community setting. In Box 2, we list factors that would support the option of home birth. Additional facilitating factors in our case included the fact that parents had had a previous home birth, lived close to the hospital (and paediatric hospice) and the infant had a condition that is typically associated with normal (or near normal) neonatal condition initially after birth.

**Box 2.** Potential criteria for considering home birth in the setting of perinatal palliative care.

1. Usual maternal criteria for supporting home birth are met 2. Fetus has a certain antenatal diagnosis and prognosis and is eligible for perinatal palliative care 3. Parents are clear in their desire for postnatal palliative care and understand that advanced life-sustaining measures will not be available immediately after birth 4. Anticipated condition of infant at birth is not likely to need immediate specialist palliative care input 5. Paediatric, palliative care and nursing teams are available to provide outpatient support after birth including symptom management 6. Experienced midwifes available to support home birth are aware of fetal condition and willing to support palliative care plan for infant

## View

Whilst an antenatal diagnosis of a life-limiting fetal condition brings immense pain, it also offers a unique opportunity to plan care in advance and to shape a baby’s arrival into the world in line with parental wishes and values. With staff shortages across maternity and neonatal care^16^ there may be challenges in providing this level of care and appropriately supporting this option may not always be possible for other families in the same situation. Nonetheless, our experience and that of Lily’s parents, indicates that home birth with perinatal palliative care is possible and can be hugely valued by families. Given the growing role of children’s hospices in perinatal palliative care^17^ future work could explore whether hospices themselves might be an alternative birth location for some families. Further research is warranted to understand the individualised birthing and postpartum preferences of women and families receiving perinatal palliative care and how these may vary between different countries. Social, cultural and resource considerations may mean that perinatal palliative care as it is currently conceptualised in HRCs may not be applicable to the needs of LMRCs^3^ and so further research is needed to delineate the role of perinatal palliative care in contexts where home birth is either more or less common. Further research to explore the safety, acceptability and feasibility of providing palliative care in the setting of home birth is needed. Consideration should also be given as to what additional support may be required for the healthcare professionals who care for families in such circumstances. Finally, there is a need to evaluate the long-term impact on families of having a home birth with perinatal palliative care support.

## Learning points for practice/research

- Further research is needed to explore the birthing and postpartum preferences of women and families receiving perinatal palliative care and how these may differ in different global contexts
- Individual healthcare organisations may benefit from multidisciplinary discussions involving obstetric, midwifery, neonatal, palliative care and hospice teams to assess whether home birth in the setting of perinatal palliative care is feasible in their context
- Further research is warranted to evaluate the impact on healthcare staff of facilitating home births alongside perinatal palliative care

## References

[1] Dombrecht L Chambaere K Beernaert K , et al Components of perinatal palliative care: an integrative review. Children 2023; 10(3): 482. 36980040 10.3390/children10030482 PMC10047326

[2] Côté-Arsenault D. The case for perinatal palliative care and expanded research. Palliat Med 2023; 37(9): 1286–1288. 37787505 10.1177/02692163231203852

[3] Abayneh M Rent S Ubuane PO , et al Perinatal palliative care in sub-Saharan Africa: recommendations for practice, future research, and guideline development. Front Pediatr 2023; 11: 1217209. 37435165 10.3389/fped.2023.1217209 PMC10331424

[4] NICE. Clinical guideline [NG61]: end of life care for infants, children and young people with life-limiting conditions: planning and management, https://www.nice.org.uk/guidance/ng61/resources/end-of-life-care-for-infants-children-and-young-people-with-lifelimiting-conditions-planning-and-management-pdf-1837568722885 (2016, accessed 10 January 2024). 28045476

[5] Office for National Statistics. Birth characteristics in England and Wales: 2021, https://www.ons.gov.uk/peoplepopulationandcommunity/birthsdeathsandmarriages/livebirths/bulletins/birthcharacteristicsinenglandandwales/latest (2023, accessed 14 September 2023).

[6] Brunton G Wahab S Sheikh H , et al Global stakeholder perspectives of home birth: a systematic scoping review. Syste Rev 2021; 10(1): 291. 10.1186/s13643-021-01837-9 PMC8561961 34727980

[7] NICE. Clinical guideline [CG190]: intrapartum care for healthy women and babies. https://www.nice.org.uk/guidance/cg190/resources/intrapartum-care-for-healthy-women-and-babies-pdf-35109866447557 (2022, accessed 14 September 2023). 31820894

[8] Committee on Fetus and Newborn, Watterberg KL Papile LA , et al Planned home birth. Pediatrics 2013; 131(5): 1016–1020.

[9] Knowles G Vente TM Fry JT. Hospice home birth. Pediatrics 2021; 147(3_MeetingAbstract): 533.

[10] Alphonso N Angelini A Barron DJ , et al Guidelines for the management of neonates and infants with hypoplastic left heart syndrome: The European Association for Cardio-Thoracic Surgery (EACTS) and the Association for European Paediatric and Congenital Cardiology (AEPC) Hypoplastic Left Heart Syndrome Guidelines Task Force. Eur J Cardiothorac Surg 2020; 58(3): 416–499. 32856064 10.1093/ejcts/ezaa188

[11] Frommelt MA. Challenges and controversies in fetal diagnosis and treatment: hypoplastic left heart syndrome. Clin Perinatol 2014; 41(4): 787–798. 25459774 10.1016/j.clp.2014.08.004

[12] Group B in EC. Perinatal and maternal outcomes by planned place of birth for healthy women with low risk pregnancies: the Birthplace in England national prospective cohort study. BMJ 2011; 343: d7400. 10.1136/bmj.d7400 PMC3223531 22117057

[13] Li Y Townend J Rowe R , et al Perinatal and maternal outcomes in planned home and obstetric unit births in women at ‘higher risk’ of complications: secondary analysis of the Birthplace national prospective cohort study. BJOG 2015; 122(5): 741–753. 25603762 10.1111/1471-0528.13283 PMC4409851

[14] Jankowski J Burcher P. Home birth of infants with congenital anomalies: a case study and ethical analysis of careproviders’ obligations. J Clin Ethics 2015; 26(1): 27–35. 25794291

[15] Sidgwick P Harrop E Kelly B , et al Fifteen-minute consultation: perinatal palliative care. Arch Dis Child 2017; 102(3): 114–116. 10.1136/archdischild-2016-310873 27849163

[16] All Party Parliamentary Groups (APPGs) on Baby Loss and Maternity. Safe staffing: the impact of staffing shortages in maternity and neonatal care, https://www.sands.org.uk/sites/default/files/Staffing%20shortages%20-%20APPG%20report,%20Oct%2022%20(final).pdf (2022, accessed 14 September 2023).

[17] Tatterton MJ Fisher MJ Storton H , et al The role of children’s hospices in perinatal palliative care and advance care planning: the results of a national British survey. J Nurs Scholarsh 2023; 55(4): 864–873. 36541193 10.1111/jnu.12866

